# Towards bridging the translational gap by improved modeling of human nociception in health and disease

**DOI:** 10.1007/s00424-022-02707-6

**Published:** 2022-06-03

**Authors:** Maximilian Zeidler, Kai K. Kummer, Michaela Kress

**Affiliations:** Institute of Physiology, Medical University of Innsbruck, Innsbruck, Austria

**Keywords:** microRNA, mRNA, Monogenetic pain disorders, Pathological pain, Neuropathic pain, Inflammatory pain

## Abstract

Despite numerous studies which have explored the pathogenesis of pain disorders in preclinical models, there is a pronounced translational gap, which is at least partially caused by differences between the human and rodent nociceptive system. An elegant way to bridge this divide is the exploitation of human-induced pluripotent stem cell (iPSC) reprogramming into human iPSC-derived nociceptors (iDNs). Several protocols were developed and optimized to model nociceptive processes in health and disease. Here we provide an overview of the different approaches and summarize the knowledge obtained from such models on pain pathologies associated with monogenetic sensory disorders so far. In addition, novel perspectives offered by increasing the complexity of the model systems further to better reflect the natural environment of nociceptive neurons by involving other cell types in 3D model systems are described.

## Introduction

The discriminative component of pain sensations includes the ability to identify a stimulus as originating from somatic or visceral tissue, to inform about the physical properties of the stimulus along a continuum of intensities, and to localize it in space and time. Of particular importance for the extraction of information on the stimulus is the primary nociceptive neuron, which serves transduction and transformation processes and transmits the information to the next level of integrating neurons in the CNS [1]. The first recordings from nociceptive nerve fibers were pioneered by Yngve Zotterman [2] and Ainsley Iggo [3] in nonhuman species followed by microneurography recordings from human nociceptive afferents by Hagbarth and Vallbo [4]. Since then, nociceptors have been classified according to conduction velocities of their axons and responsiveness to specific natural physical, chemical, and electrical stimuli. Sir Charles Sherrington introduced these important functions as the main tasks of nociceptors [5]. In addition, it is generally accepted that nociceptors act as chemosensors for cytokines, prostaglandins, kinins, bioactive sphingolipids, and other mediators or chemical irritants [6, 7]. Based on these findings, the concept of Sherrington has been questioned, and the idea emerged that these primary afferents, rather than functioning simply as Sherringtonian nociceptors, may have more general and long-term roles in signaling and more recently even in controlling the microenvironment and components of the immune system in their respective target tissue [8–10]. Specifically, peptidergic nociceptors release neuropeptides in response to noxious stimuli not only by volume transmission to modify spinal pain circuits but also at their peripheral terminals in a reaction causing vasodilation and plasma extravasation in their target tissue, which has been long known as axon reflex (alias neurogenic inflammation, Lewis reaction – [11–13]). Of the eleven different sensory neuron subtypes emerging from recent unbiased transcriptomic studies [14], peptidergic and non-peptidergic small neurons serve different roles: peptidergic nociceptors control specific immune cell types but non-peptidergic neurons are equally important for balancing of the local immune reaction via the release of the fast neurotransmitter glutamate from nerve terminals in their target tissues [15, 16].

To date, nociceptor excitation and sensitization processes have mainly been explored and seminal findings made in nonhuman model systems, but increasing numbers of studies are exploiting the human dorsal root ganglion (DRG) as a model system [17–21]. The studies available to date, however, suggest fundamental differences between mice and men. In particular, there are human nociceptor populations that are not found in mice [21]. Also, transcripts for transient receptor potential channels, cholinergic receptors, potassium channels, sodium channels, and other markers/targets are differentially expressed, suggesting human-specific spatial and functional organization of neuronal cell subpopulations and supporting the idea that sensory system organizational principles are different between both species [22].

A major drawback, however, is the restricted availability of live human nociceptive neurons for transcriptomic and functional in vitro studies for ethical and logistic reasons. The results from nonhuman models turn out to be only partially transferable to healthy humans and even less to patients suffering from pain disorders arising from nociceptor pathologies [23]. Primary cultures of human or mouse DRG neurons also suffer from drawbacks as they adopt a neuropathic phenotype under culture conditions [24]. To better elaborate on human nociceptor (patho)physiology towards patient-centered mechanistic insight and the development of mechanism-based treatments, rigidly controlled human model systems are largely favorable over nonhuman models and offer major advantages to develop personalized medicines tailored to the individual needs of each patient. This review article compares nociceptor phenotypes in mice and humans and genetic pain disorders and summarizes additional cellular components interacting with nociceptors as well as recent approaches towards improved modeling of human nociception to provide an overview on the current state of the art and identify methodological needs.

## Exploring molecular sensory neuron phenotypes

The cell bodies of primary afferent neurons serving different functionalities are located within dorsal root ganglia and trigeminal ganglia (TG). In general, large diameter neurons give rise to low-threshold mechanosensitive fibers whereas small diameter neurons are slowly conducting, unmyelinated C-fibers or thinly myelinated Aδ-fibers implicated in thermosensation or the transduction of noxious stimuli. Immunoreactivity to specific cell markers is generally used to distinguish these neuron classes since, for example, neurofilament heavy chain (NEFH) is abundant in large, myelinated neurons, and the intermediate filament protein type 3 (peripherin) is typically found in small diameter sensory neurons [25–27]. These small diameter sensory neurons are further subclassified into C-fiber nociceptors expressing the high-affinity neurotropic growth factor (NGF) receptor TrkA, Aδ-fiber nociceptors expressing the TrkB receptor for brain-derived neurotrophic factor (BDNF), and mechanoreceptors or pruriceptors expressing TrkC for neurotrophin-3 (NT-3) [28]. While the expression of TrkA in nociceptors is highly correlated with a peptidergic sensory neuron phenotype, the absence of TrkA and the expression of the Runt-related transcription factor-1 (RUNX1) and RET more likely drive sensory neurons towards a non-peptidergic fate [29]. Electrophysiological classification of nociceptors is usually based on the presence of TTX-sensitive and resistant voltage-gated sodium channels or endogenous temperature transducers belonging to the class of transient receptor potential channels (TRP channels) [30–37]. These classifications were the basis for a broad range of studies in rodents investigating the different roles and distinct synaptic connections in the spinal dorsal horn of peptidergic nociceptors that express neuropeptides such as calcitonin gene–related peptide (CGRP)/somatostatin (SST) or substance P (TAC1) and non-peptidergic nociceptors that bind the isolectin B4 [38–42]. Although sensory neurons largely share transcriptional signatures, specific molecular genetic profiles and a high-resolution atlas of sensory neuron subtypes illustrate cell-type–specific functionalities such as mechanoreceptors expressing TrkB, the two-pore potassium channel KCNK4, and the mechanotransducer channel PIEZO2 or proprioceptors expressing TrkC and parvalbumin (PVALB) [43, 44].

Bulk RNA sequencing and bulk proteomics reveal a strong overlap of DRG developmental transcription factors such as PRDM12, BRN3A, and DRGX, suggesting conserved developmental signatures of sensory neurons between humans and rodents [45, 46]. However, with the recent rise of single-cell RNA (scRNA) sequencing technologies, increasing evidence documents that even nociceptor subtypes are much more complex than initially thought: Five clusters of larger diameter sensory neurons are tied together by the expression of NEFH and separated by the abundance of TrkC, TrkB, PVALB, and RET, revealing mechanoreceptors (TrkB^high^, RET^high^) as well as proprioceptors (TrkC^high^, Pvalb^high^) [14]. In addition, two subclusters of peptidergic sensory neurons and three subclusters of non-peptidergic neurons can be distinguished by the unique transcriptomic signatures of the P2X purinoceptor 3 (P2RX3, non-peptidergic nociceptors) and tachykinin precursor 1 (TAC1, peptidergic nociceptors) [14]. More recent studies further increased the resolution of cell type clustering by enhancing the read coverage, adding different species (i.e., primates), and extending the number of cells as well as adding disease phenotypes such as neuropathic pain, which led to more refined clusters of sensory neurons [47–49]. These subclusters are defined by the expression of SST and PVALB, the nociceptor-specific voltage-gated sodium channels Na_v_1.7 (SCN9A) and Na_v_1.8 (SCN10A), and the unique expression pattern of cytokine receptors such as IL31RA [49, 50]. Thus, 11 different clusters with unique gene expression signatures were associated with mechano-heat sensory neurons, which are predominantly distinguished by the expression of the neuropeptide galanin (GAL), claudin-9 (CLDN9), zinc finger CCHC-type containing 12 (ZCCHC12), lysophosphatidic acid receptor 2 (LPAR2), TRPM8 or TRPA1, and mechanoceptive markers such as PIEZO2, KCNK2, as well as MRGPRB4.

**Fig. 1:**
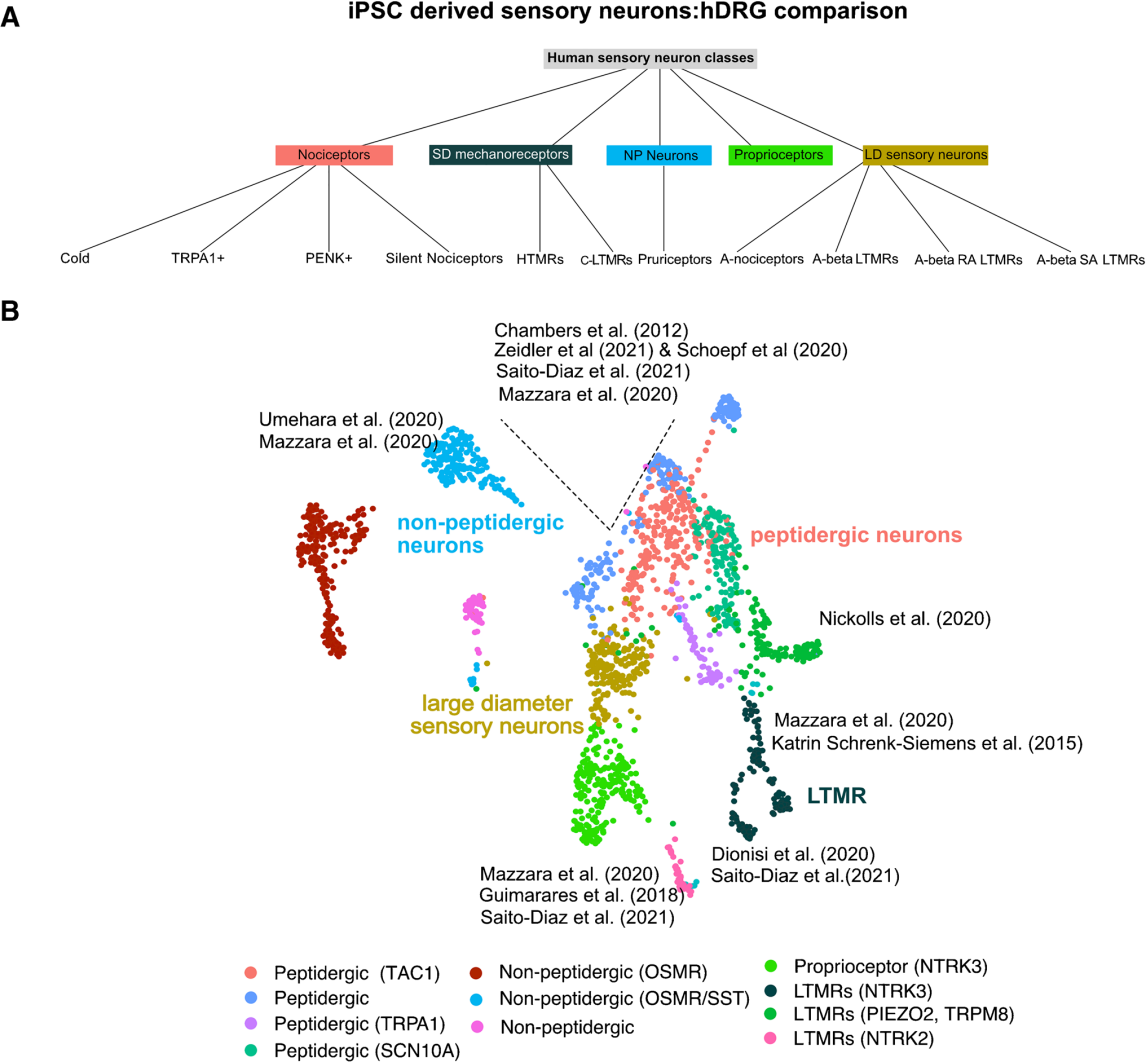
**A** Tree view of hDRG sensory neuron subpopulations, derived from Tavares-Ferreira et al. [52]. **B** Mapping of iPSC-derived sensory neuron equivalents to hDRG single-nuclei data derived from Nguyens (2021)

In contrast to the extensive literature on rodent nociceptors, to date only two scRNA transcriptomic studies address human DRG (hDRG) neurons [51, 52]. In general, expression signatures and cell type clusters appear badly conserved between rodents, nonhuman primates, and humans, and several neuronal subpopulations were identified (Fig. 1A, [47, 52]). For example, the most abundant marker NEFH used to distinguish rodent small diameter nociceptors from larger diameter sensory neurons is gradually expressed in the majority of hDRG sensory neurons and cannot be used as a reliable discriminator in human DRGs [22, 51, 53, 54]. To overcome this hurdle, recent advances in the field of spatial transcriptomics allow for a more precise correlation of cell soma sizes with RNA expression signatures, thereby efficiently separating small from large-diameter sensory neurons [52]. Cell clustering and marker expression suggest that the majority of hDRG sensory neurons adopt a peptidergic phenotype, and non-peptidergic human DRG neurons expressing the oncostatin M receptor (OSMR) and SST are implicated in the perception of itch rather than pain [51]. Five nociceptor-related neuron types can be distinguished from non-nociceptive neurons based on the conserved expression of TrkA and Na_v_1.8 [51]. Human nociceptors responding to different modalities such as cold or heat show specific expression of the marker molecules TRPA1, proenkephalin (PENK), TRPM8, the alpha 3 subunit of nicotinic acetylcholine receptor (CHRNA3), or TrkC^+^ nociceptors. Species differences were especially evident for putative silent nociceptors that are usually activated following inflammation [52].

## Human-derived sensory neuron differentiation

Since acquisition of human sensory neuron tissue is challenging and mostly relies on postmortem material from elderly patients, research started to adopt recent advances in induced pluripotent stem cell (iPSC) models. Human iPSCs are generated from somatic cells such as fibroblasts obtained from skin biopsies or blood samples [55–57]. Since their discovery, the Yamanaka factors Oct4, SOX2, c-Myc, and KLF4/NANOG are used to initialize the reprogramming of somatic cells into iPSCs that exhibit pluripotent properties [56]. This was the initial kickoff to establish and develop differentiation protocols that enable the differentiation of iPSCs into virtually every cell type, such as cardiomyocytes, immune cells, as well as neurons [58, 59].

In contrast to cortical neurons, differentiation protocols for sensory neurons were established relatively late [60–63]. Induced sensory neurons are established via intermediate neural crest cells, which express SOX9, SNAIL, and dHAND [64, 65]. Subsequent studies extended these protocols by selectively blocking activin/TGF-β and BMP signaling, also called dual-SMAD inhibition, and cells that express markers comparable to mouse neural crest cells (which can differentiate into sensory neuron-like cells), such as the SRY-box transcription factor 9/10 (SOX9, SOX10) and the paired box 3 gene (PAX3), which are established efficiently [60, 66]. In 2012, Chambers et al. identified a set of three small molecule inhibitors (CHIR99021, SU5402, and DAPT,called 3i for “three inhibitors”) that efficiently induce sensory neuron-like cell differentiation following dual-SMAD inhibition [61]. While the administration of CHIR99021 is indispensable for the differentiation process, SU5402 and DAPT enhance the efficiency and the speed of the differentiation process [61]. The resulting “mature” differentiated cell types are positive for POU domain class 4 transcription factor 1 (POU4F1, BRN3A) and islet-1 (ISL1) and exhibit neuronal morphology indicated by neurite outgrowth and synapse formation [67, 68]. In addition, TrkA and neurogenin-1 (NEUROG1) suggest peripheral neuronal induction and expression of Na_v_1.7, Na_v_1.8, and TRPV1 specialization into nociceptor-like sensory neurons at D12 [61, 68]. Maturation of neurons is induced by co-administration of the nerve growth factor (NGF), the glial cell–derived neurotrophic factor (GDNF), BDNF, and NT-3. These more mature iPSC-derived sensory neurons fire single as well as trains of action potentials and are immune-positive for the peptide hormone precursors TAC1 (substance P, neurokinin A, neuropeptide K, and neuropeptide gamma) and CALCA (CGRP, calcitonin, and katacalcin), indicating that they adopt a peptidergic sensory neuron phenotype [61, 68, 69].

Although this protocol is considered the “gold standard” for iPSC-derived sensory neuron and nociceptor differentiation, several studies introduced modifications by inhibiting 3i until day 12 instead of day 10 of differentiation, replating cells before passaging, neglecting NT-3 as growth factor, adding ascorbic acid, changing media gradients, or adding proliferation inhibitors (such as AraC or MitoC), which overall can lead to an increased efficiency of differentiation [69–71]. Different timings of 3i inhibition, changes in growth factor administration (NGF, BDNF, GDNF, and NT-3), and addition of BMP-4 or cAMP produce different subtypes of sensory neurons such as proprioceptors, mechanoreceptors, as well as pruriceptors [72–74] (for an overview of the available differentiation protocols, see Table 1). Neuronal induction media composed of DMEM, N2, NEAA, and heparin, followed by administration of retinoic acid and BMP4, successfully induce TrkA^+^ nociceptors from human embryonic stem cells [75]. However, the effect on the cell phenotype of these adjustments has not yet been sufficiently resolved.

**Table 1:** Differentiation protocols of iPSC-derived sensory neurons

Cell type	Differentiation initiation	Differentiation	Maturation	Sensory neuron subtype	Publication	Notes
iPSC	SB431542(D0-D5) LDN192189(D0-D5)	CHIR990021 SU5402 DAPT	NGF BDNF GDNF (NT-3) (cAMP, ascorbic acid)	Peptidergic nociceptors	Chambers et al. [61]	Minor adjustments in, e.g., Young et al. [68] Meets et al. (2019) Pettingill et al. [71] Schoepf et al. [67]
MEFs/HEFs		BRN3A NEUROG1/NEUROG2	NGF BDNF GDNF	Mature sensory neurons	Blanchard et al. (2014)	
smNPCs		CHIR990021 SU5402 DAPT BMP4 (DAY4)	dbcAMP GDNF BNDF NGF	LTMR	Zhu et al	
iPSC	SB431542(D0-D5) LDN192189(D0-D5)	CHIR990021 (D2-D7) SU5402 (D2-D8) DAPT (D2-D8)	NGF GDNF BDNF NT-3	Proprioceptor	Dionisi et al. [72]	
iPSC	Noggin(D0-D10) SB431542(D0-D10)	SB431542(D11-D18) EGF(D11-D18) FGF(D11-D18)	BDNF GDNF NGF NT-3 ascorbic acid cAMP	Puriceptor	Umehara et al. [74]	
hESC	Retinoic acid BMP4	NEUROG1	NGF GDNF GDF-7 IGF-1	Nociceptors	Boisvert et al. [75]	
iPSC	SB431542 bFGF/EGF	BRN3A NEUROG2	BDNF GDNF NT-3 NGF	C-LTMR	Nickolls et al. [77]	
iPSC	SB431542(D0-D10) LDN192189(D0-D10)	CHIR990021 SU5402 DAPT	BDNF GDNF NGF NT3 ascorbic acid	Nociceptors Proprioceptors Mechanoceptors	Mazzare et al. (2020)	Differentiation in low-adhesion 96-well plates
iPSC	CHIR99021 (D0-2) BMP(D0-D2) Y-27632(D0-2)	SB431542(D2-12) CHIR99021(D2-D12)	NGF GDNF BDNF NT-3 DAPT retinoic acid (RA)	Nociceptors Proprioceptors Mechanoreceptors	Saito-Diaz et al. [78]	Immunopanning to retrieve different sensory neuron subtypes
hESC	Sphere Medium, FGF EGF	RA NGF BDNF GDNF NT-3	*Differentiation	Mechanoreceptors	Katrin Schrenk-Siemens et al. (2015)	Sphere medium consists of DMEM/F12, neurobasal medium, B27, N2, glutamine, insulin

In addition, alternative approaches are emerging such as trans-differentiation to generate TrkA^+^/CGRP^+^ peptidergic sensory neurons from human and mouse embryonic fibroblasts overexpressing a fusion construct of BRN3A with neurgenin-1/2 [76] or to differentiate human iPSCs into mechanoreceptors [77]. A chemically defined differentiation strategy followed by immunopanning invokes differentiation into functional nociceptors, proprioceptors, and mechanoceptors simultaneously [78]. Even iPSC-derived DRG organoids can be generated with iPSCs differentiating into sensory nociceptors, mechanoreceptors, and proprioceptors [79].

## Mapping iDN phenotypes to the landscape of human DRGs

Although iPSC-derived sensory neurons are increasingly used as a model system, phenotypic characterization of the retrieved cells and comparison to human sensory neurons are only insufficiently explored. Recently published hDRG scRNA datasets offer the opportunity to phenotype the iPSC-derived neurons against expression data derived from human DRG sensory neuron subpopulations (Fig. 1A, [52]). Mapping signatures of marker genes such as neuropeptides (CGRP, TAC1, SST), voltage-gated sodium channels (Na_v_1.7, Nav_1_.8), TRP channels (TRPV1, TRPM8, TRPA1), and transcription factors (DRGX, MAF) suggest that most iPSC-derived nociceptor-like neurons represent an immature state of peptidergic nociceptors that express substance P, Na_v_1.8, TRPV1, NEFH, and peripherin but also the P2X-purinoreceptor 3, which is a common marker for non-peptidergic sensory neurons (Fig. 1, [22, 67, 69]). In addition, a trajectory analysis of all relevant and enriched human transcription factors (DRGX, PIRT, BRN3A, and TLX3) reveals that most of these factors are also enriched in iPSC-derived sensory neurons and abundant in human peptidergic sensory neurons. Furthermore, iPSC-derived nociceptor-like neurons transcriptionally approach to hDRG throughout differentiation [69]. Likewise, iPSC-derived low-threshold mechanoreceptors (LTMRs), proprioceptors, as well as pruriceptors can be mapped to their human DRG orthologs, due to the abundant expression of TrkC and TrkB. While proprioceptors show strong expression of TrkC, mechanoreceptors are either positive for TrkC, TrkB, parvalbumin, and the transcription factor short stature homeobox 2 (SHOX2) [72]. iPSC-derived pruriceptor expression of itch-related molecules such as IL31R, IL-4R, and the histamine receptor HRH1 is highly correlated with non-peptidergic itch-related human DRG neurons [74].

In general, it is difficult to estimate the affiliation of different iPSC-derived sensory neurons to specific sensory neuron clusters, since these cells represent an immature developmental state in contrast to mature human DRG sensory neurons and only a minor fraction of nociceptor-like neurons are truly mature [80] [79]. Thus, there is a considerable need for protocols to improve the maturity state of iPSC-derived sensory neurons, as already shown for cortical neurons [81]. This further suggests the necessity for more comparative studies between hDRGs and iPSC-derived sensory neurons, to elucidate the actual maturity state of the derived neurons as well as meta-analyses and deconvolution studies to compare the different iPSC-derived sensory neuron subtypes and identify differences as well as similarities with hDRG sensory neurons.

## Modeling hereditary disorders affecting nociceptors

With the increase of routinely performed genomic diagnostic testing of patients, our knowledge about hereditary disorders affecting nociceptors has enormously increased in the last decades. Not only have different hereditary sensory neuropathies been described based on the particular clinical symptom presentation, but also their genetic predisposition has been revealed for the majority of them [82]. Likewise, familial hemiplegic subtypes of migraine – a common primary headache disorder that presents with moderate to severe unilateral pain attacks with several typical variants with or without aura – are associated with specific disease-associated mutations in the voltage-gated calcium channel CACNA1A (FHM1), the sodium/potassium-transporting ATPase subunit ATP1A2 (FHM2), and the voltage-gated sodium channel SCN1A (FHM3) genes [83]. This knowledge about disease-causing mutations in the affected genes opens new possibilities to investigate the underlying pathogenetic mechanisms and to generate personalized treatment strategies based on patient-derived iPSC models (Table 2).

**Table 2:** Monogenetic sensory neuropathies

Disease term	Abbreviation	MedGen UID	Genes^*^	References
Neuropathy, hereditary sensory and autonomic, type IA	HSAN1A	1,716,450	SPTLC1 (9q22.31)	[121]
Neuropathy, hereditary sensory and autonomic, type IB, with cough and gastroesophageal reflux	HSN1B, HSAN1B	330,880	HSN1B (3p24-p22)	–
Hereditary sensory and autonomic neuropathy, type IC	HSAN1C	462,246	SPTLC2 (14q24.3)	None
Hereditary sensory neuropathy, type ID	HSN1D	462,322	ATL1 (14q22.1)	[128]
Hereditary sensory and autonomic neuropathy, type IE	HSN1E	481,515	DNMT1 (19p13.2)	[129]
Hereditary sensory neuropathy, type IF	HSN1F	816,524	ATL3 (11q13.1)	None
Hereditary sensory and autonomic neuropathy, type IIA	HSAN2A	416,701	a.KIF1A (2q37.3) b.RETREG1 (5p15.1) c.SCN9A (2q24.3) d.WNK1 (12p13.33)	a.[130] b.None c.[70, 101] d.None
Hereditary sensory and autonomic neuropathy, type IIB	HSAN2B	413,474	RETREG1 (5p15.1)	None
Hereditary sensory and autonomic neuropathy, type IIC	HSAN2C	481,798	KIF1A (2q37.3)	[130]
Neuropathy, hereditary sensory and autonomic, type IID	HSAN2D	860,491	SCN9A [131]	[70, 101]
Hereditary sensory and autonomic neuropathy, type III; familial dysautonomia	HSAN3	41,678	ELP1 (9q31.3)	[132–135]
Hereditary sensory and autonomic neuropathy, type IV; CIPA, congenital insensitivity to pain and anhidrosis	HSAN4	6915	NTRK1 (1q23.1)	[136]
Hereditary sensory and autonomic neuropathy, type V; congenital sensory neuropathy with selective loss of small myelinated fibers	HSAN5	6916	NGF (1p13.2)	[137]
Neuropathy, hereditary sensory and autonomic, type VI	HSAN6	761,278	DST (6p12.1)	[138]
Neuropathy, hereditary sensory and autonomic, type VII; congenital insensitivity to pain with hyperhidrosis and gastrointestinal dysfunction	HSAN7	816,212	SCN11A (3p22.2)	None
Neuropathy, hereditary sensory and autonomic, type VIII	HSAN8	894,363	PRDM12 (9q34.12)	None
Neuropathy, hereditary sensory and autonomic, type IX; spastic paraplegia 49, autosomal recessive	HSAN9	762,260	TECPR2 (14q32.31)	None
Indifference to pain, congenital, autosomal recessive; channelopathy-associated	CIP	344,563	SCN9A (2q24.3)	[101]
Primary erythromelalgia	–	8688	SCN9A (2q24.3)	[70]
Paroxysmal extreme pain disorder	PEPD	331,565	SCN9A (2q24.3)	None
Charcot-Marie-Tooth disease, type 2B	CMT2B	371,512	RAB7A (3q21.3)	[139]
Familial hemiplegic migraine, type 1	FHM1	331,388	CACNA1A (19p13.13)	[92, 93]
Familial hemiplegic migraine, type 2	FHM2	355,962	ATP1A2 (1q23.2)	none
Familial hemiplegic migraine, type 3	FHM3	400,655	SCN1A (2q24.3)	none
Migraine, with or without aura 13	MGR13	462,258	KCNK18 (10q25.3)	[71]

HSN, hereditary sensory neuropathy; HSAN, hereditary sensory and autonomic neuropathy; CIP, congenital indifference to pain; CIPA, congenital insensitivity to pain and anhydrosis; FHM, familial hemiplegic migraine; MGR, migraine. ^*^ Associated genes were extracted from the *MedGen* Database

Currently, nine different subtypes of hereditary sensory neuropathies (HSN; if the autonomic nervous system is involved: HSAN; 1–9) are listed in the National Center for Biotechnology Information (NCBI) database MedGen for human medical genetics (NCBI 2022; [84]. They are generally classified based on age of onset, clinical features, type of inheritance, and genetic background [82, 85]. To date, iPSC-derived nociceptor models offer particularly promising opportunities to explore personalized pharmacological treatments for those HSNs with a hypernormal pain phenotype such as gain-of-function mutations in the SCN9A gene, coding for the DRG, and sympathetic neuron-specific voltage-gated sodium channel Na_v_1.7 [86, 87].

Primary erythromelalgia patients suffer from unbearable pain episodes and redness, mainly in the distal extremities, impaired distal temperature sensation, and itching. This is caused by a gain-of-function mutation of SCN9A [88]. Erythromelalgia patient–derived nociceptors have recently been generated that exhibit decreased firing thresholds together with a hyperpolarizing shift of Na_v_ activation as compared to control neurons [70]. This model system will be helpful for further investigations of the disease-causing mechanisms as well as for the screening of potential treatment-effective compounds [89].

Paroxysmal extreme pain disorder (PEPD) is similar to erythromelalgia also caused by a gain-of-function mutation of SCN9A. Symptoms include skin redness and flushing as well as severe pain attacks in different parts of the body [90]. To date, no iPSC models for PEPD-specific SCN9A mutations are available.

Familial hemiplegic migraine 1 (FHM1) is in the majority of cases caused by a missense mutation of the CACNA1A gene that codes for the pore-forming subunit of the neuronal voltage-gated calcium channel Ca_v_2.1, leading to gain-of-function effects that result in hyperexcitability [83, 91]. No studies on iPSC-derived neurons from FHM1 patients have been performed yet. Nevertheless, a number iPSC model systems derived from a different patient group of spinocerebellar ataxia 6, which is based on loss-of-function mutations in the CACNA1A gene, were generated and in part functionally investigated [92–94, 94, 95, 95].

Familial hemiplegic migraine 2 (FHM2) has been associated with mutations in the ATP1A2 gene that encodes a catalytic subunit of the Na^+^/K^+^-ATPase ion transport pump [83]. It is therefore generally important for the regulation of electrochemical gradients across cell membranes but specifically relevant for the function of excitable cells, including neurons. No functional investigations of patient-derived iPSCs carrying ATP1A2 mutations are available to date.

Familial hemiplegic migraine 3 (FHM3) is caused by mutations in the voltage-gated sodium channel gene SCN1A coding for Na_v_1.1. This ion channel is important for the sodium ion permeability of excitable membranes and was shown to be mainly expressed in inhibitory GABAergic interneurons [83]. Due to this prominent role in the modulation of network excitability, Na_v_1.1 is also highly associated with different epilepsy syndromes. And while in epilepsies, the associated mutations mainly lead to loss-of-function effects provoking seizure activity, FHM3 mutations usually cause gain-of-function effects. Therefore, functional investigation of the plethora of iPSC-derived model systems (e.g., [96–98]) from epilepsy patients can only partly be used to decipher the mechanisms behind migraine pain.

Migraine with or without aura 13 (MGR13) has been directly linked to a specific gene, the potassium channel KCNK18. This gene codes for the two-pore potassium channel TRESK and is important for regulating the excitability of neurons in the pain pathway [83]. Pettingill et al. [71] could show in nociceptors differentiated from patient-derived iPSCs that a CRISPR-Cas9–mediated repair of a TRESK frameshift mutation normalized neuronal excitability. This and a few other studies point toward exciting novel possibilities for gene therapeutic treatments of neuropathies associated with nociceptor dysfunction, which are currently still in their infancy.

## Modeling complex pathologies

**Fig. 2:**
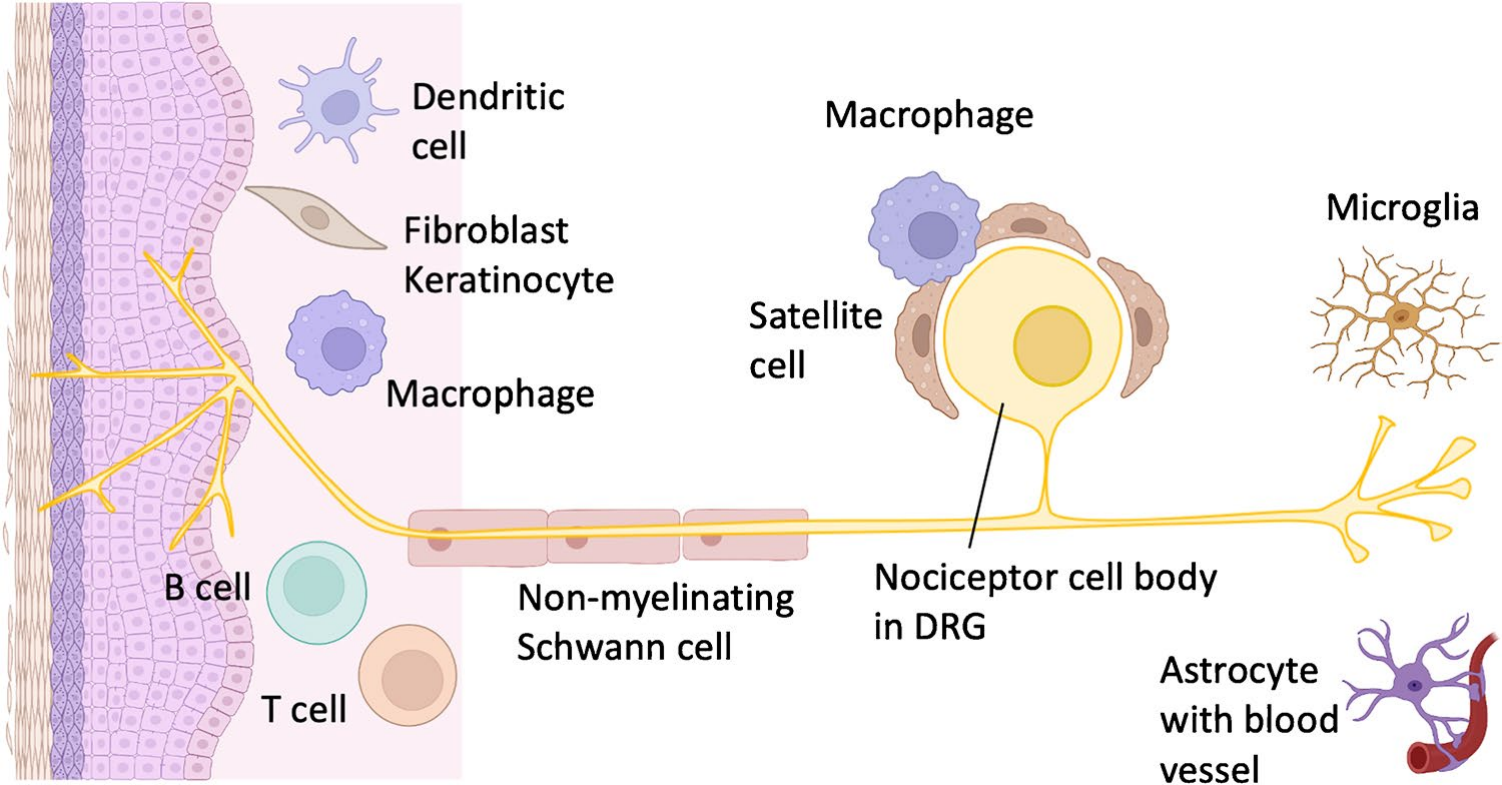
Nociceptors and associated cells in the target tissue (skin, left), DRG, and the spinal cord (right) to be implemented in complex model systems (generated with BioRender®)

Healthy nociceptors serve important roles as an important alarm system and in host defense by sensing physical and chemical stimuli including noxious heat, cold, pressure, and danger signals [99]. Nociceptive transduction, transformation, or transmission may be affected by monogenetic pathologies, and these can be sufficiently modeled in nociceptors derived from patient iPSC clones [70, 86, 100, 101]. However, the pathogenesis of acquired pain disorders such as painful arthritis, complex regional pain syndrome (CRPS), or postherpetic neuralgia is more complex, and numerous studies have reported the contribution of immune and glia cells to nociceptor dysfunction in preclinical models of pathological pain. For example, inflammatory pain is characterized by immune processes at the nociceptor nerve terminal, which liberates a multitude of different pro-inflammatory mediators [102]. Although the different nociceptor subtypes and even more each individual nociceptor expresses only a certain subset of membrane receptors and ion channels, the nociceptor population as a whole possesses the full equipment to sense these mediators and react to their presence with the discharge of action potentials or by lowering their activation thresholds for physical stimuli [14, 48, 103]. Since Elspeth McLachlan and her team first published on immune cells invading the space between sensory neuron cell bodies and the covering satellite cell layer in the DRG as a consequence of peripheral nerve injury, neuroimmune processes have emerged as critical components for inflammatory and even neuropathic pain disorders [102, 104, 105]. Neuroimmune processes are active following peripheral nerve injuries, and the neuroimmune triad contributes to the generation of neuropathic pain at the lesion site but also at non-injured parts of the axon and even the DRG or the central process connecting the nociceptor to spinal projection neurons [104, 106–108]. Modeling the complexity of the nociceptor neuron in 3D organoid models employing human iPSC-derived nociceptors therefore needs to include relevant components reflecting its healthy or diseased environment by co-culturing with relevant interactors at the peripheral terminal, the axon, DRG, as well as the central terminal in the spinal cord such as keratinocytes, macrophages, glia cells, and other types of immune cells (Fig. 2).

### Skin and other target organs

Starting from a 3D platform of human iPSCs-derived nociceptors for peripheral nerve modeling and tissue innervation with heterologous Schwann cells, organoid models engineered from human cells only are now becoming available, which employ sensory neurons and Schwann cells differentiated in parallel even from the same iPSC donor together with engineered skin [109–111]. In the skin, specific non-neuronal cell types are crucial for nociceptor function such as keratinocytes releasing ATP [111–114] and enterochromaffin cells closely collaborating with nociceptive primary afferents in the gut by the exchange of chemical signals for chemosensation [115]. Within target tissues, the communication between target cells and nociceptors is bilateral whereby nociceptors sense danger signals on the one hand and regulate immunity on the other for example in the lung as well as in the skin. This immunomodulation is mediated by different types of afferents through the release of glutamate or the neuropeptide CGRP [10, 116].

### Schwann cells and satellite cells

Bidirectional signaling between axons and the peripheral nerve glia Schwann cells is essential for both the development and maintenance of sensory neuron morphology and function. However, primary human Schwann cells are challenging as a resource for nerve tissue engineering (for a review, see [117]). Co-cultures of iPSC-derived sensory neurons with myelinating Schwann cells were first developed to explore mechanisms of demyelination and neurodegeneration underlying respective disorders such as Guillain–Barre-Syndrome and remyelination therapies [118–120]. First, human iPSC–derived sensory neurons were cultured with rat Schwann cells and produced long-term and stable myelinating co-cultures, which developed specialized domains of axonal interaction with myelinating Schwann cells, such as clustered voltage-gated sodium channels at the node of Ranvier and Shaker-type potassium channels (K_v_1.2) at the juxtaparanode. Several regulators of myelination were explored in these models such as BACE1 or NRN1 and may be relevant for the function of myelinated nociceptors of the A-fiber type or even nonmyelinating Schwann cells covering C-fiber nociceptive axons [120, 121]. However, specific models for directed iPSC differentiation into human nonmyelinating Schwann cells and co-cultivation with human nociceptive neurons are currently unavailable and modeling the role of Schwann cells for nociceptor function has even received less attention.

### Immune cells (macrophages, lymphocytes)

Mutual neuroimmune cross talk thus appears to be important for maintaining tissues such as skin or the gut in a healthy state. Nociceptors are important regulators of resident immune cells in the skin by balancing their activity through the release of neuropeptides and glutamate [16]. However, a disbalance of pro- and anti-inflammatory components characterizes disorders leading to inflammatory or neuropathic pain with respective alterations of nociceptor functions. In particular, neuropathic pain shows relevant features of a neuroimmune disorder and involves not only neuronal components, but also Schwann cells and satellite cells, different cell types of the peripheral immune system at the lesion site or in the DRG, as well as spinal microglia and astrocytes (for a review, see [122]). More recently, more painful disorders with neuroimmune pathologies are emerging. For example, fibromyalgia, which has been enigmatic until very recently, has a striking autoimmune component and metabolic disturbances that can be explored in human model systems [123, 124]. Co-cultivation of iPSC-derived nociceptors with relevant immune cells of the same donor may help to model the causative changes on a more personalized basis for monogenetic disorders or even more complex pathologies.

## Concluding remarks

Human nociceptors are different from rodents such as they show specific signatures of gene expression and neuropeptides that are typically found in the majority of native human nociceptors such as CGRP or substance P [110, 125]. These differences are partially preserved in nociceptive neurons derived from human iPSCs [14, 52, 69], and these can be exploited for human-centered mechanistic studies and patient-targeted drug discovery in particular for monogenetic pain disorders, which, however, provide benefit only for a minor subgroup of chronic pain patients.

Furthermore, complex 3D systems model human nociceptors in a more precise manner and their natural environment. Based on these considerations and in light of the individual genetic background, all relevant cell types within the 3D model are to be obtained from the same donor. This approach precisely constructs a platform to explore individual nociceptive processes, and the new organoid models offer intriguing possibilities to study the pathophysiology of nociceptors together with their environment and further develop them into screening assays for novel analgesic pharmacies and even personalized treatments.

However, such organoid models are less easily accessible to controlled read-out systems due to their increased complexity and 3D structure. Neuropeptide release evoked by depolarizing concentrations of KCl or irritants such as capsaicin can serve as a quantitative read-out measure of nociceptor excitation [110, 126]. In addition, individual iPSC-derived cell types are accessible to genetic modifications using CRISPR/Cas9 or introduction of Cre recombinase under respective driver constructs. Optogenetic tools as well as reporter dyes can be introduced into the individual cellular components before implementing them in the engineered organoid. Recent developments of specifically designed multi-electrode array chips for 3D organoids will help to overcome the challenges [127].

Overall, specifically tailored co-cultivation of all relevant cell types as an engineered model for particular disorders such as neuropathic pain, fibromyalgia, or even inflammatory bowel disease offers major advantages for a better understanding of mutual interaction of the involved cells with nociceptors and can be exploited for analgesic drug discovery purposes [89]. Modeling human nociception, in particular by reprogramming patient-derived iPSCs into complex organoids involving all relevant cell types, thus rises great expectations to overcome the translational gap between preclinical research and unmet clinical needs for effective analgesic pharmaceuticals.

## Data Availability

Not applicable.
